# Complete genome sequence of *Dehalobacter restrictus* PER-K23^T^

**DOI:** 10.4056/sigs.3787426

**Published:** 2013-07-30

**Authors:** Thomas Kruse, Julien Maillard, Lynne Goodwin, Tanja Woyke, Hazuki Teshima, David Bruce, Chris Detter, Roxanne Tapia, Cliff Han, Marcel Huntemann, Chia-Lin Wei, James Han, Amy Chen, Nikos Kyrpides, Ernest Szeto, Victor Markowitz, Natalia Ivanova, Ioanna Pagani, Amrita Pati, Sam Pitluck, Matt Nolan, Christof Holliger, Hauke Smidt

**Affiliations:** 1 Wageningen University, Agrotechnology and Food Sciences, Laboratory of Microbiology, Dreijenplein 10, NL-6703 HB Wageningen, The Netherlands.; 2 Ecole Polytechnique Fédérale de Lausanne (EPFL), School of Architecture, Civil and Environmental Engineering, Laboratory for Environmental Biotechnology, Station 6, CH-1015 Lausanne, Switzerland.; 3 DOE Joint Genome Institute, Walnut Creek, California, USA; 4 Los Alamos National Laboratory, Bioscience Division, Los Alamos, New Mexico, USA

**Keywords:** *Dehalobacter restrictus* type strain, anaerobe, organohalide respiration, PCE, TCE, reductive dehalogenases

## Abstract

*Dehalobacter restrictus* strain PER-K23 (DSM 9455) is the type strain of the species *Dehalobacter restrictus*. *D. restrictus* strain PER-K23 grows by organohalide respiration, coupling the oxidation of H_2_ to the reductive dechlorination of tetra- or trichloroethene. Growth has not been observed with any other electron donor or acceptor, nor has fermentative growth been shown. Here we introduce the first full genome of a pure culture within the genus *Dehalobacter*. The 2,943,336 bp long genome contains 2,826 protein coding and 82 RNA genes, including 5 16S rRNA genes. Interestingly, the genome contains 25 predicted reductive dehalogenase genes, the majority of which appear to be full length. The reductive dehalogenase genes are mainly located in two clusters, suggesting a much larger potential for organohalide respiration than previously anticipated.

## Introduction

*Dehalobacter restrictus* strain PER-K23 (DSM 9455), is the type strain of the species *Dehalobacter restrictus* [1]. Currently two pure cultures of *D. restrictus* have been described, namely *D. restrictus* strains PER-K23 and TEA [1,2].

We publish here the first full genome of a pure culture within the genus *Dehalobacter* and a preliminary comparison with a previously obtained metagenome from a co-culture containing *Dehalobacter sp.* strain E1 and *Sedimentibacter* sp [3].

Organohalide respiration (OHR) is considered as a key process in bioremediation of sites contaminated with organohalides such as tetrachloroethene (PCE) and trichloroethene (TCE), leading to a great interest in understanding the physiology and metabolism of organohalide respiring bacteria (OHRB). Most OHRBs are facultative organohalide respirers, capable of dehalogenating a limited number of halogenated compounds, as part of a versatile metabolism. This group consists of several genera, including *Desulfitobacterium*, *Geobacter* and *Sulfurospirillum*. Other isolates are obligate OHRB, among which isolates and enrichments of different *Dehalococcoides mccartyi* strains are the best studied. They have been shown to degrade a large variety of halogenated compounds solely using H_2_ as the electron donor. Until recently, the genus *Dehalobacter* had been thought to encompass exclusively obligate OHRB, however, at least some members of this genus have been described as able to ferment dichloromethane [4,5]. *D. restrictus* strain PER-K23 is an obligate OHRB, and like *Dehalococcoides mccartyi*, uses H_2_ as a sole electron donor. These similarities in physiology and ecology are noteworthy since *Dehalobacter* spp. are phylogenetically closely related to the metabolically versatile *Desulfitobacterium* spp.

*D. restrictus* strain PER-K23 was isolated from a packed bed column containing sediment from the river Rhine collected near Wageningen, the Netherlands, and granular sludge from a sugar refinery. This column had been fed with PCE for a prolonged period, prior to isolation of *D. restrictus* strain PER-K23 [6].

*D. restrictus* strain PER-K23 was chosen for genome sequencing because it is the type strain of the *Dehalobacter restrictus* species. Studying the genome gives an improved insight into the physiology and evolution of the genus *Dehalobacter* and may ultimately lead to unlocking its full potential for bioremediation.

## Classification and features

**Figure 1:**
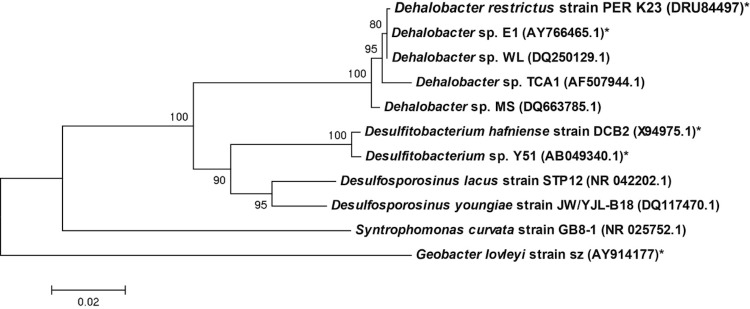
Phylogenetic tree highlighting the position of *Dehalobacter restrictus* relative to phylogenetically closely related organisms. 16S rRNA sequences were retrieved from Genbank (NCBI), and accession numbers are given in parentheses. Strains from which a full genome sequence are available are indicated with an asterisk. Phylogenetic analysis was done using the MEGA5 software package [20]. Sequences were aligned using the MUSCLE algorithm before a neighbor joining tree was constructed and validated with 1,000 bootstraps [21,22]. The reference bar indicates 2% sequence divergence.

*Dehalobacter restrictus* is a member of the phylum *Firmicutes*, class *Clostridia,* order *Clostridiales,* and family *Peptococcaceae* [1],(Table 1). *D. restrictus* is closely related to the newly sequenced *Dehalobacter sp.* strain E1 [3], but grows in pure culture. Both *Dehalobacter* spp. and *Desulfitobacterium* spp. belong to the family *Peptococcaceae* (Figure 1). All members of this family are anaerobes, constituting a diverse group with respect to their metabolism and morphology [23]. *D. restrictus* strain PER-K23 is a rod-shaped bacterium with a single lateral flagellum and has not been reported to form spores. It stains Gram-negative, even though it phylogenetically belongs to the Gram-positive *Firmicutes*, and does not have an outer membrane, indicating that it should be considered a Gram-positive [1]. *D. restrictus* strain PER-K23 grows by coupling the oxidation of H_2_ to the reduction of PCE or TCE, growth has not been observed with any other electron donor or acceptor, nor has fermentative growth been shown [1,6]. *D. restrictus* strain PER-K23 requires iron as a trace element, the vitamins thiamine and cyanocobalamin, and the amino acids arginine, histidine and threonine for growth [1].

**Table 1:** Classification and general features of *D. restrictus* strain PER-K23 according to MIGS recommendations [7].

**MIGS ID**	**Property**	**Term**	**Evidence code** ^a^
		Domain *Bacteria*	TAS [8]
		Phylum *Firmicutes*	TAS [9-11]
		Class *Clostridia*	TAS [12,13]
	Current classification	Order *Clostridiales*	TAS [14,15]
		Family *Peptococcaceae*	TAS [14,16]
		Genus *Dehalobacter*	TAS [17,18]
		Species *Dehalobacter restrictus*	TAS [17,18]
		Type strain PER-K23	
	Gram stain	Negative	TAS [1]
	Cell shape	Straight rod	TAS [1]
	Motility	Motile	TAS [1]
	Sporulation	Not observed	TAS [1]
	Temperature range	10-37 °C	TAS [1]
	Optimum temperature	25-30 °C	TAS [1]
	Carbon source	Acetate, yeast extract	TAS [1]
	Energy source	H_2_ as sole electron donor	TAS [1]
	Terminal electron receptor	PCE and TCE	TAS [1]
MIGS-6	Habitat	Anaerobic river sediment	TAS [1,6]
MIGS-6.3	Salinity	Not tested	
MIGS-22	Oxygen	Strictly anaerobic	[1,6]
MIGS-15	Biotic relationship	Free living	[1]
MIGS-14	Pathogenicity	None known	
MIGS-4	Geographic location	River Rhine, near Wageningen, The Netherlands	[1,6]
MIGS-5	Sample collection time	1992	

^a^ Evidence codes - IDA: Inferred from Direct Assay; TAS: Traceable Author Statement (i.e., a direct report exists in the literature); NAS: Non-traceable Author Statement (i.e., not directly observed for the living, isolated sample, but based on a generally accepted property for the species, or anecdotal evidence). These evidence codes are from the Gene Ontology project [19].

### Genome project history

Table 2 presents the project information in compliance to MIGS version 2.0 [24].

**Table 2:** Project information

**MIGS ID**	**Property**	**Term**
MIGS-31	Finishing quality	Closed genome
MIGS-28	Libraries used	Two genomic libraries, one paired-end 454 library and one Illumina library.
MIGS-29	Sequencing platforms	454 GS FLX Titanium and Illumina GAii
MIGS-31.2	Fold coverage	8.5 for 454 and 120 for Illumina
MIGS-30	Assemblers	Newbler version 2.3, VELVET, version 1.0.13 and phrap, version SPS – 4.24
MIGS-32	Gene calling method	Prodigal, GenePRIMP
	Genome Database release	December 28^th^, 2011
	Genbank ID	PRJNA66209
	Genbank Date of Release	
	GOLD ID	Gi05571
	Project relevance	Type strain, Bioremediation, Biotechnology

### Growth conditions and DNA isolation

*Dehalobacter restrictus* strain PER-K23, DSM9455, was cultivated anaerobically as previously described [1]. DNA was extracted from bacterial pellets using the protocol recommended by the JGI. In brief, cell walls were digested with lysozyme before DNA was purified with hexadecyltrimethylammonium bromide, phenol and chloroform, and precipitated with isopropanol. Quality and quantity of the obtained DNA were checked by running aliquots on agarose gels using lambda phage DNA as mass standard and HindIII digested lambda phage DNA as a size marker.

### Genome sequencing and assembly

The draft genome of *Dehalobacter restrictus* PER-K23 was generated at the DOE Joint genome Institute (JGI) using a combination of Illumina [25], and 454 technologies [26]. For this, genome we constructed and sequenced an Illumina GAii shotgun library which generated 77,929,756 reads totaling 5,922.7 Mb, and 1 paired end 454 library with an average insert size of 10 kb which generated 318,117 reads totaling 59.3 Mb of 454 data. All general aspects of library construction and sequencing performed at the JGI can be found at the JGI website [27]. The initial draft assembly contained 90 contigs in 1 scaffold. The 454 paired end data were assembled together with Newbler, version 2.3-PreRelease-6/30/2009. The Newbler consensus sequences were computationally shredded into 2 kb overlapping fake reads (shreds). Illumina sequencing data was assembled with VELVET, version 1.0.13 [28], and the consensus sequence were computationally shredded into 1.5 kb overlapping fake reads (shreds). We integrated the 454 Newbler consensus shreds, the Illumina VELVET consensus shreds and the read pairs in the 454 paired end library using parallel phrap, version SPS - 4.24 (High Performance Software, LLC). The software Consed [29-31] was used in the following finishing process. Illumina data was used to correct potential base errors and increase consensus quality using the software Polisher developed at JGI (Alla Lapidus, unpublished). Possible mis-assemblies were corrected using gapResolution (Cliff Han, unpublished), Dupfinisher [32], or sequencing cloned bridging PCR fragments with subcloning. Gaps between contigs were closed by editing in Consed, by PCR and by Bubble PCR (J-F Cheng, unpublished) primer walks. A total of 134 additional reactions were necessary to close gaps and to raise the quality of the finished sequence. The total size of the genome is 2,943,336 bp and the final assembly is based on 24.6 Mb of 454 draft data which provides an average 8.5× coverage of the genome and 348 Mb of Illumina draft data which provides an average 120× coverage of the genome.

### Genome annotation

Genes of *D. restrictus* strain PER-K23 were identified using Prodigal [33] as part of the Oak Ridge National Laboratory genome annotation pipeline, followed by a round of manual curation using the JGI GenePRIMP pipeline [34]. The predicted CDSs were translated and used to search the National Center for Biotechnology Information (NCBI) non-redundant database, UniProt, TIGRFam, Pfam, PRIAM, KEGG, COG, and InterPro databases. These data sources were combined to assert a product description for each predicted protein. Non-coding DNA and miscellaneous features were predicted using tRNAscan-SE [35], RNAMMer [36], Rfam [37], TMHMM [38], and signalP [39].

## Genome properties

The genome consists of a single chromosome with a total size of 2,943,336 bp with 45% G+C content. A total of 2,908 genes were predicted, 2,826 of which are protein-coding genes. Genes with putative function corresponded to 76.7% (2,168), of all protein coding sequences with the remaining annotated as hypothetical proteins. In addition, 1,174 protein coding genes belong to 356 paralogous families in this genome. The properties and the statistics of the genome are summarized in Tables 3,4 and 5.

**Table 3:** Nucleotide content and gene count levels of the genome

**Attribute**	Value	% of total^a^
Genome size (bp)	2,943,336	100.00
DNA coding region (bp)	2,473,591	84.04
DNA G+C content (bp)	1,311,589	44.56
Total genes^b^	2,908	100.00
RNA genes	82	2.82
Protein-coding genes	2,826	97.18
Genes in paralog clusters	1,174	40.37
Genes assigned to COGs	2,127	73.14
Genes with signal peptides	756	26.00
Genes with transmembrane helices	755	25.96
Paralogous groups	356	40.37
Reductive dehalogenases^c^	25	0.86

^a^ The total is based on either the size of the genome in base pairs or the total number of protein coding genes in the annotated genome.

^b^ Also includes 143 pseudogenes.

^c^ Including pseudogenes

**Table 4:** Number of genes associated with the general COG functional categories

**Code**	**Value**	**%age**	**Description**
J	149	6.4	Translation
A	--	--	RNA processing and modification
K	176	7.6	Transcription
L	180	7.8	Replication, recombination and repair
B	1	0.1	Chromatin structure and dynamics
D	44	1.9	Cell cycle control, cell division, chromosome partitioning
Y	--	--	Nuclear structure
V	39	1.7	Defense mechanisms
T	166	7.2	Signal transduction mechanisms
M	148	6.4	Cell wall/membrane biogenesis
N	71	3.1	Cell motility
Z	--	--	Cytoskeleton
W	--	--	Extracellular structures
U	73	3.1	Intracellular trafficking and secretion
O	84	3.6	Posttranslational modification, protein turnover, chaperones
C	169	7.3	Energy production and conversion
G	64	2.8	Carbohydrate transport and metabolism
E	166	7.2	Amino acid transport and metabolism
F	59	2.5	Nucleotide transport and metabolism
H	118	5.1	Coenzyme transport and metabolism
I	40	1.7	Lipid transport and metabolism
P	105	4.5	Inorganic ion transport and metabolism
Q	27	1.2	Secondary metabolites biosynthesis, transport and catabolism
R	241	10.4	General function prediction only
S	203	8.7	Function unknown
-	781	26.9^a^	Not in COGs

^a^ Percentage of the total number of protein coding genes in the annotated genome.

**Table 5:** Reductive dehalogenase paralogs encoded in the genome of *D. restrictus* strain PER-K23

**Locus tag ^a^**	**Ortholog in *Dehalobacter sp.*** **strain E1 ^b^**	**Comment**
Dehre_0785	No	
Dehre_0793	No	
Dehre_0806	No	
Dehre_0808	No	N-terminally truncated
Dehre_0815	No	N-terminally truncated
Dehre_0820	No	
Dehre_0826	No	
Dehre_0830	No	
Dehre_0832	(98.7; Dhb965)	
Dehre_0835	(98.7; Dhb968)	
Dehre_0990	(100; Dhb84)	
Dehre_1408	(99.6; Dhb1133)	
Dehre_2012	(96.6; Dhb1238)	C-terminally truncated^c^
Dehre_2022	No	
Dehre_2026	No	
Dehre_2031	No	
Dehre_2037	No	
Dehre_2039	No	
Dehre_2044	No	
Dehre_2052	No	
Dehre_2058	No	C-terminally truncated
Dehre_2064	No	
Dehre_2065	No	
Dehre_2398	(90.4; Dhb490)	PceA ^d^
Dehre_2792	No^e^	Partial sequence

^a^ RdhA paralogs are listed in order of their position in the genome. Light grey indicates RdhA paralogs belonging to *rdh* cluster A (Dehre_785-835) and dark grey *rdh* cluster B (Dehre_2012-2065).

^b^ Orthology defined as more than 90% pairwise identity at the amino acid level, as suggested in [40]. Identity percentage based on full length RDHs and locus tag of the corresponding genes in *Dehalobacter sp.* strain E1 are given in brackets [3]. Identity percentages were calculated using MatGat [41].

^c^ For the comparison, a manually curated version of Dehre_2012 was used, i.e. the entire gene without the annotated frame-shift mutation.

^d^ Dehre_2398 corresponds to the biochemically characterized PCE reductive dehalogenase (PceA) [42].

^e^ The sequence is conserved between the two strains, but no gene is annotated at this position in D. sp. Strain E1.

## Insights from genome sequencing

### Reductive dehalogenase paralogs

The genome of *D. restrictus* contains 25 loci predicted to code for proteins with sequence homology to reductive dehalogenases (RDHs). Among these 25 genes, one is a partial sequence and four are truncated due to possible frame-shift mutations (Table 5). This high number is in contrast to those found to date for metabolically versatile organohalide respirers. These possess a limited number of RDHs typically in the range of 1 to7 [43,44]. The number of RDHs in *D. restrictus* lies in the same range as seen in specialized organohalide respirers, such as *Dehalococcoides mccartyi* strains and *Dehalogenimonas lykanthroporepellens*, which have been predicted to possess between 10 and up to 36 RDHs [45,46].

For *D. restrictus* however, this finding is intriguing since, PCE and TCE, currently, are the only electron acceptors known to be utilized by strain PER-K23 [1]. The identification of a total of 25 *rdhA* genes suggests that *D. restrictus* possesses a much larger potential for OHR metabolism, than previously anticipated.

**Figure 2:**
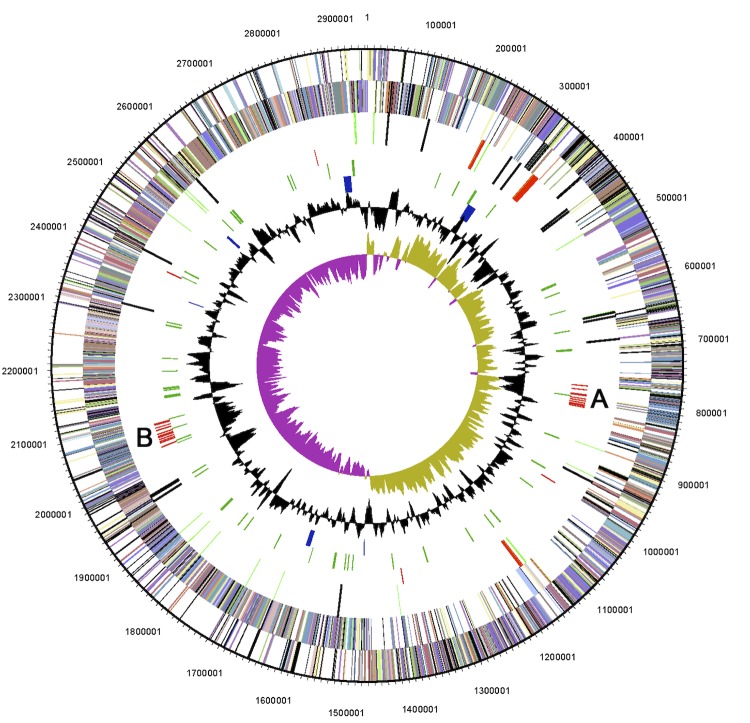
Circular map of the chromosome of *D. restrictus* strain PER-K23. Labeling from the outside circle towards the inside circle. Numbers outside the map indicate nucleotide positions; Circles 1 and 2: predicted coding sequences, including pseudogenes, on the forward and reverse strand, respectively (colored by COG categories); Circle 3: RNA genes (tRNAs green, rRNAs red, other RNAs black); Circle 4: Position of reductive dehalogenase genes, in red, both functional and truncated, A and B indicate two *rdh* clusters; Circle 5: Position of transposases including inactive derivatives, in green; Circle 6: Position of genes related to corrinoid synthesis and uptake, in blue; Circle 7: GC content (peaks out/inside the circle indicate above or below average GC content, respectively: Circle 8: GC skew, calculated as (G-C)/(G+C), purple or olive indicates values lower or higher than 1, respectively.

The majority of the *rdhA* genes are located in two clusters, one on each chromosome arm, with all but two RDHs being encoded on the leading strand. Cluster A is approximately 54 kb long, located on the right chromosome arm and contains 10 reductive dehalogenase genes including two truncated ones. Cluster B is approximately 61 kb long, located on the left chromosome arm and contains 11 reductive dehalogenase genes, of which two appear truncated (Table 5 & Figure 2).

The remaining three complete RDH genes and one partial RDH encoding gene are scattered throughout the genome (Table 5 & Figure 2). A similar pattern has previously been observed in the genomes of *Dehalococcoides mccartyi* strains, where the majority of the RDHs are located on each side of, and close to the origin of replication [47]. These regions were described as high plasticity regions, where frequent events of rearrangement and horizontal gene transfer are thought to occur. It was suggested that these regions enable fast adaptation to dehalogenation of new organohalides, while at the same time protecting key metabolic functions from being disrupted by horizontal gene transfer events [47].

We identified transcriptional regulators of the CRP/FNR type being encoded by genes in the vicinity of most of the RDH encoding genes, with PceA (encoded by Dehre_2398) as a notable exception [48]. A regulator of this type has been demonstrated to regulate the expression of the genes that code for chlorophenol reductive dehalogenase (*cpr* operon in *Desulfitobacterium dehalogenans* and *Desulfitobacterium hafniense* strain DCB-2 [49]. The presence of transcriptional regulator genes close to almost all *rdhA* genes suggest that their transcription is regulated. This was confirmed by a recent study looking at transcription of *rdh* genes and the proteome of *Dehalobacter restrictus* strain PER-K23 growing in the presence of H_2_ and PCE. In this study we found that PceA (encoded by Dehre_2398) was highly present at both RNA and proteomic level, whereas the remaining RDHs and the corresponding transcripts were either not detected at all or at very low levels, suggesting that the RDH encoding genes are tightly regulated, and probably only expressed in the presence of their specific substrate [48].

Recently the draft genome of *Dehalobacter sp.* strain E1 was published [3]. This genome contains nine potentially functional *rdhA* genes, and one pseudogene. Six of these are conserved between *D. restrictus* strain PER-K23 and strain E1 (Table 5). Two of the conserved *rdhA* genes are located at the edge of cluster A and one at the edge of cluster B. Interestingly all four *rdhA* genes present outside cluster A or B are conserved between the two strains, which may indicate that both cluster A and B represent high plasticity regions unique to *D. restrictus* (Table 5). Currently, *pceA* (encoded by Dehre_2398) is the only RDH-encoding gene from *Dehalobacter restrictus* to be characterized in detail. The corresponding gene product PceA has been shown to catalyze the reduction of PCE to TCE and TCE to *cis*-DCE, the only two electron acceptors demonstrated to support growth of *D. restrictus* [1,42]. The *pceA* gene belongs to a gene cluster, *pceABCT* (Dehre_2395-2398), which is highly similar to a gene cluster identified in a composite transposon structure identified in several *Desulfitobacterium* strains [50-52]. The transposon structure is not conserved in *D. restrictus* although the gene cluster is flanked by sequences resembling transposase genes in a late state of decay (Dehre_2394 and 2399). This combined with the fact that the *pceABCT* gene cluster including the cryptic transposases and the surrounding genomic context are conserved between *D. restrictus* and *D*. strain E1 (data not shown) suggest that the presence of *pceABCT* is the result of an ancient horizontal gene transfer event.

### Corrinoid synthesis and uptake

Corrinoid is the key cofactor in characterized RD catalytic subunits. *Dehalobacter restrictus* strain PER-K23 requires vitamin B_12_ in the medium for growth [1].

Therefore it is noteworthy to report the presence of a full set of corrinoid biosynthesis genes in the genome of *D. restrictus*, although *cbiH* (Dehre_2856) encoding precorrin-3B C17-methyltransferase displays a frame-shift mutation, and consequently is annotated as a pseudogene. The vitamin B_12_ synthesis pathway is encoded by two distinct gene clusters in *D. restrictus* strain PER-K23, where Dehre_2848-2865 encode enzymes of the upper pathway, and Dehre_1606-1615 the lower pathway. One additional gene (Dehre_1488) belonging to the lower pathway is located elsewhere in the genome (Figure 2) [48]. The genome encodes several gene clusters associated with corrinoid uptake and salvaging pathways. Preliminary studies of the proteome from cultures grown at standard conditions or with partial vitamin B_12_ depletion showed that gene products encoded by one of the salvaging pathways (Dehre_0281-0291) were much more abundant in the vitamin B_12_ starved cells than in the cells grown under standard concentrations (J. Maillard and T. Kruse unpublished data). These findings suggest that the *de novo* corrinoid synthesis pathway is not functional and that *Dehalobacter restrictus* strain PER-K23 is dependent on salvaging corrinoids from the environment.

### Hydrogenases

Another interesting feature is the presence of genes predicted to code for eight different hydrogenases. These include three periplasmic membrane-bound Ni/Fe uptake hydrogenases, consisting of three subunits: a catalytic unit, an Fe/S cluster protein and a membrane-bound *b*-type cytochrome (Dehre_551-553, 1061-1063 and 2405-2007), two six-subunits membrane-bound energy-conserving Ni/Fe hydrogenases (Dehre_1568-1573 and 1645-1650), and three Fe-only hydrogenases (Dehre_1739-1741, 2317-2320 and 2372-2374). The Fe-only hydrogenases consist of the catalytic subunit and two to three putative electron transferring subunits.

The presence of multiple uptake hydrogenases has also been observed in *Desulfitobacterium* spp., whereas *Dehalococcoides mccartyi* strains only have one uptake hydrogenase [43,44,53]. The two six-subunits Ni/Fe resemble the Hyc and Ech complexes found in *Dehalococcoides mccartyi* strain 195 [54], as well as the Hyc complex found in *Desulfitobacterium* spp [43,44,55].

Disrupting either one uptake hydrogenase or the six-subunits energy-conserving hydrogenase in *Desulfitobacterium dehalogenans* led to loss of the ability to grow using lactate or formate as electron donor and 3-chloro-4-hydroxyphenylacetate as electron acceptor, indicating that hydrogenases may play an important role in the electron transport chain to RD catalytic subunits, even when hydrogen is not used as the initial electron donor [55].

The role of the six-subunit hydrogenase complexes are still poorly understood. It has been speculated that they play a role in generating low potential electrons for OHR by reverse electron flow. However, this was considered as unlikely in one study where *Dehalococcoides mccartyi* strain 195 was cultivated in the presence of varying concentrations of hydrogen [56]. The exact role of the different hydrogenases in *Dehalobacter restrictus* strain PER-K23 still needs further studies.

The genome also encodes an intact Wood-Ljungdahl pathway (Dehre_0130-0155 and 2348-2351). The presence of a whole or partial Wood-Ljungdahl pathway has been observed in other OHRB. The closely related *Desulfitobacterium hafniense* strains Y51 and DCB-2 both contain genes predicted to encode a full Wood-Ljungdahl pathway, and strain DCB-2 has been shown to fix CO_2_ [43,44]. The more distantly related *Dehalococcoides mccartyi* strains have been shown to contain partial Wood-Ljungdahl pathways, but its exact role in the metabolism of these organisms remains unclear [57,58].

The genome of *D. restrictus* contains 72 genes annotated as encoding transposases or inactive derivatives thereof, whereas it only contains few phage-associated genes despite the lack of a CRISPR phage immunity system.

Cells of *Dehalobacter restrictus* strain PER-K23 are motile [1]. The genome contains genes for synthesis of flagella and several genes predicted to be involved in chemotaxis. The role of chemotaxis in OHRB is currently understudied. Chemotactic behavior towards metals has been described for *Geobacter*, some members of this genus have been shown to be OHRB. Chemotactic behavior towards organohalides has, however, not been described for *Geobacter* spp [59-61].

## Conclusion

The presence of an unexpectedly large number of putative RDH encoding genes suggests a far larger potential for use in bioremediation than previously anticipated, especially if *Dehalobacter restrictus* strain PER-K23 is attracted by organohalides in a chemotactic manner. The complete genome sequence of *Dehalobacter restrictus* strain PER-K23, the type strain of the genus *Dehalobacter*, represents a significant leap towards understanding the physiology, ecology and evolution of this specialized organohalide respiring group of bacteria. Current work focuses on obtaining a deeper understanding of the expression and regulation of the RDH genes, and thereby expanding the known organohalide substrate range of this organism. Shot-gun proteome analysis will aid in deciphering the metabolism of *D. restrictus* strain PER-K23 and allow generation of refined genome scale metabolic models of these dedicated degraders.
